# Bi-directional and multivariate mendelian randomization analysis of the relationship between circulating 25-hydroxyvitamin D concentration and obstructive sleep apnea

**DOI:** 10.1186/s12890-022-02172-y

**Published:** 2022-10-14

**Authors:** Xi Luo, Ruijing Chang, Jianli Zhang, Peng Jiang, Sicheng Xu

**Affiliations:** 1 Department of Respiratory, General Hospital of Xinjiang Military Region, 830000 Urumqi, China; 2 Department of Respiratory Intensive Care Unit, the First Affiliated Hospital of Xinjiang Medical University, 830054 Urumqi, China

**Keywords:** Obstructive sleep apnea, Mendelian randomization, 25-hydroxyvitamin D, Causality

## Abstract

**Background:**

25-hydroxyvitamin D [25(OH)D] deficiency in patients with Obstructive Sleep Apnea (OSA) has long been noted, but identifying the exact causal relationship remains hard. Investigation of the causality between 25(OH)D deficiency and OSA would help facilitate disease prevention.

**Methods:**

We conducted a two-sample bi-directional Mendelian randomization (MR) study. For forward analysis, 237 newly identified genetic variants are used as proxies for 25(OH)D to estimate the unconfounded effect on OSA among 16,761 OSA cases and 201,194 controls of European ancestry. Reverse analysis was performed to detect the causal impact of OSA on 25(OH)D levels. The inverse variance weighted (IVW) method was used as the primary analysis. Sensitivity analysis was performed to evaluate the robustness of our results. Multivariate MR analysis was conducted to evaluate the direct link between 25(OH)D and OSA after accounting for body mass index (BMI).

**Results:**

IVW indicated that OSA causally associated with a lower level of 25(OH)D ((β = -0.03, 95% CI = -0.06 ~ -0.007, *P* = 0.01). No evidence of the causal link from 25(OH)D to OSA was detected (OR = 0.99, 95% CI = 0.88 ~ 1.12, *P* = 0.85). Sensitivity analysis suggested the MR estimates were not biased. Multivariate MR analysis indicated the effect of OSA on 25(OH)D vanished upon accounting for BMI (β = -0.011, 95% CI = -0.028 ~ 0.007, *P* = 0.23).

**Conclusion:**

This MR study provided evidence that OSA was causally associated with a lower level of 25(OH)D, which might be driven by BMI. Obesity management should be enhanced in patients with OSA to prevent 25(OH)D deficiency.

**Supplementary Information:**

The online version contains supplementary material available at 10.1186/s12890-022-02172-y.

## Introduction

Obstructive sleep apnea (OSA) refers to a kind of sleep-related disorder characterized by a repetitive, partial, or complete obstruction of the upper respiratory tract during sleep, resulting in an intermittent drop in blood oxygen partial pressure, blood oxygen saturation, and hypercapnia[1]. Epidemiological research has reported that OSA prevalence among the population aged 30 to 69 years is rising in all countries around the world, which accounts for 14% of the world’s population[2]. Daytime drowsiness, weariness, inattention, memory loss, or headaches, are more frequent in patients with OSA, which all exert a negative influence on life quality and expectancy[3]. Besides, OSA patients are more likely to have cardiovascular, cerebral, and pulmonary vascular problems, as well as systemic multisystem pathophysiological alterations[4, 5]. Considering the high prevalence and detrimental consequences of OSA, it’s critical to investigate the risk factors of OSA to facilitate OSA screening and prevention.

Vitamin D, the liposoluble vitamin, has two forms: D2 (ergocalciferol, obtained through food sources) and D3 (cholecalciferol, produced inside the skin when it is exposed to ultraviolet light, or 25(OH)D[6]. Vitamin D3 [25(OH)D] has been proposed to be associated with the health conditions of multiple systems, including urinary[7], endocrine[8], and blood systems [9]. As a sleep-related disorder, OSA has also been linked with 25(OH)D in the existing literature[10–12]. Such a link has aroused researchers’ curiosity, as it would support the rationale for investigating dietary 25(OH)D supplementation for OSA prevention. However, the existing evidence about the association between 25(OH)D and OSA is based on conventional observational studies, making residual confounding and reverse causality unable to be ruled out. Moreover, owing to the enormous cost and time, it is impractical to perform randomized controlled trials (RCTs). As such, the causality between 25(OH)D and OSA remains unclear.

Mendelian randomization (MR) is a valuable method for causality inference via human genetics because of its initial application in evaluating if low cholesterol results in cancer[13–16]. Given that genetic variants are randomly assigned in advance based on parental genotype, an MR design can exclude the confounding effect of the surrounding environment. Consequently, single nucleotide polymorphisms (SNPs) can serve as instrumental variables (IVs) to evaluate the causal impact of exposures on outcomes of interest. Additionally, large open-source genome-wide association study (GWAS) summary statistics provide ideal datasets to precisely examine the bi-directional causal effect at a low cost. Hence, this study conducted a bi-directional MR by employing genetic instruments to explore the causal relationship between 25(OH)D and OSA.

## Materials and methods

### Study design

We conducted a bidirectional two-sample Mendelian randomization study to appraise the causal association between circulating 25(OH)D levels and OSA. MR design is based on three critical fundamental assumptions: (a) genetic instruments are robustly associated with exposure of interest (usually *P* < 5 × 10^− 8^); (b) genetic instruments are not associated with potential confounders; and (c) genetic instruments only affect the outcome through the exposure of interest. Forward [from 25(OH)D to OSA] and reverse [from OSA to 25(OH)D] MR analyses were performed. Rigorous sensitivity analysis was applied in this study to evaluate any violation of the MR assumptions.

### Data source

We obtained the GWAS data for circulating 25(OH)D from the IEU consortium (https://gwas.mrcieu.ac.uk/), which comprised 441,291 individuals of European ancestry. 16,668,957 SNPs were tested.

Summary-level statistic for OSA was obtained from the FinnGen consortium (Round 5), comprising 16,761 OSA cases and 201,194 controls (~ 63% male). Full OSA GWAS was publicly available from the FinnGen research project (https://www.finngen.fi/en), which aims to evaluate genotype-phenotype correlations to ultimately identify novel therapeutic targets. Genotype imputation, quality control and principal component analysis were undertaken in the FinnGen GWAS. The diagnosis of OSA was based on the International Statistical Classification of Diseases (ICD) codes, including ICD-10: G47.3 and ICD‐9: 3472 A. Specifically, cases with OSA were determined through subjective symptoms, clinical examination and sleep registration applying apnea‐hypopnea index (AHI) ≥ 5 per hour or respiratory event index (REI) ≥ 5 per hour. A total of 16,380,465 SNPs were tested in the OSA GWAS.

### Instrument selection

For forward analysis using 25(OH)D as the exposure, we conducted rigorous SNPs filtration. Firstly, we extracted significant independent SNPs [*P* < 5 × 10^− 8^, linkage disequilibrium (LD) r ^2^ < 0.01 within a 5,000 kb distance] from the full GWAS data to serve as the instruments proxying circulating 25(OH)D levels. Secondly, given that body mass index (BMI) is the established risk factor for OSA [17], we looked over the Phenoscanner V2 website (http://www.phenoscanner.medschl.cam.ac.uk/) to identify and exclude any exposure-SNPs associated with BMI. Thirdly, we calculated the F statistics to evaluate the strength of the SNPs, and SNPs with F < 10 were discarded. Calculation of F statistics has been described elsewhere[18]. Fourthly, we extracted the exposure-SNPs from the OSA outcome data. We replaced instruments that were unavailable in outcome data with proxy SNPs (LD r^2^ ≥ 0.8). Fifthly, we harmonized the exposure-SNPs with outcome-SNPs to exclude those being palindromic or incompatible. The SNPs left after the aforementioned filtration steps were finally used for MR analysis. For reverse analysis, instrument selection was the same as the forward analysis.

### Statistical analysis

To investigate the causality between 25(OH)D and OSA, the random-effect inverse variance weighted (IVW) method was utilized as the primary analysis in both forward and reverse MR analysis. IVW was regarded as the method with great power, of which the causal estimate was obtained by combining the Wald ratios of each SNP. Owing to bidirectional tests, the significant threshold was set at *P* < 0.025 according to Bonferroni correction (0.05/2). Estimates with *P* < 0.05 but over 0.025 were regarded as nominal significant.

To evaluate the robustness of the MR estimates, we also compared the IVW estimation with other MR models, including maximum likelihood, weighted median, and MR-Egger regression. Briefly, the weighted median method assumes that nearly half of the SNPs were invalid, whereas the MR-Egger method assumes that all the instruments were invalid. Consistent direction and magnitude across three methods enhanced the robustness of the causal inference.

We further conducted sensitivity analyses to appraise whether the observed causality was biased. We performed the Cochran Q test to evaluate heterogeneity. We also used the funnel plot to visualize heterogeneity. We then utilized the intercept term derived from MR-Egger to assess horizontal pleiotropy. Finally, leave-one-out analysis was applied to identify potential outliers that strongly bias the pooled IVW estimates.

Given that obesity has been identified as the major risk factor for OSA and that univariate MR analysis only detects the total effect of exposure on outcome, whether the causal association between 25(OH)D and OSA was direct or driven by BMI remains elucidating. Multivariate MR (MVMR) analysis was thereby implemented in our study to detect the direct link between 25(OH)D and OSA. Before MVMR analysis, we firstly evaluated the causality between 25(OH)D and BMI in both forward and reverse directions. Once the causality was detected, the role of BMI in driving the causality between 25(OH)D and OSA should be taken into account. Besides, we evaluated the causality between BMI and OSA to ensure a definite link between them despite the involvement of obesity in OSA has been well-documented. Finally, MVMR was conducted to detect whether there is a direct link between 25(OH)D and OSA after accounting for BMI using data from the MRC-IEU consortium (Table 1).

**Table 1:** Detailed information of data sources in the present study

Phenotypes	Consortium	Ancestry	Sample size	Websites
25(OH)D	IEU	European	441,291	https://gwas.mrcieu.ac.uk/
BMI	IEU	European	461,460	https://gwas.mrcieu.ac.uk/
OSA	FinnGen	European	217,955	https://www.finngen.fi/en

BMI, body mass index; OSA, obstructive sleep apnea

All analyses were performed using the “TwoSampleMR” packages (version 0.5.6) [19] and the “MendelianRandomization” package (version 0.6.0) in the R software (version 4.0.5).

## Results

**Fig. 1:**
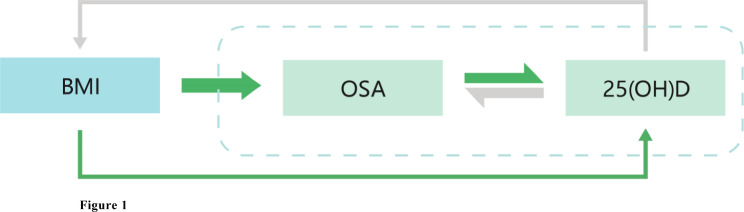
Diagram of the present Mendelian randomization study The grey arrows indicated no evidence of causal effects whereas the green arrows indicated that causalities were detected. BMI, body mass index; OSA, obstructive sleep apnea

Through rigorous instrument selection, 237 SNPs associated with 25(OH)D were used for forward MR analysis, and six SNPs associated with OSA were used for reverse MR analysis. The F statistics ranged from 25 to 445, suggesting that no weak instruments were included in our study. Basic characteristics and summary effect estimates of included variants on 25(OH)D and OSA are presented in Table S1 and Table S2. The study diagram was presented in Fig. 1. The results of the MR analyses are shown in ​Figure S1 ~ S4 and Table 2.

**Table 2:** Summarized results of bi-directional Mendelian randomization study on vitamin D and obstructive sleep apnea

MR Approaches	Vitamin D-OSA		OSA-vitamin D		OSA-vitamin D^*^	
	OR (95%CI)	*P*	β (95% CI)	*P*	β (95% CI)	*P*
MR-Egger	0.91(0.74, 1.11)	0.35	-0.04(-0.21, 0.12)	0.63	-0.05(-0.17, 0.06)	0.41
Weighted median	0.92(0.76, 1.13)	0.45	-0.02(-0.05, 0.005)	0.10	-0.03(-0.06, 0.001)	0.06
IVW	0.99(0.88, 1.12)	0.85	-0.02(-0.05, 0.01)	0.22	-0.03(-0.06, -0.01)	0.01
Maximum likelihood	0.99(0.88, 1,11)	0.84	-0.02(-0.04, 0.003)	0.09	-0.03(-0.06, -0.007)	0.01

^*^Analysis after discarding rs10928560

### Forward analysis

Using IVW, we identified a null causal effect of 25(OH)D on OSA [odds ratio (OR) = 0.99, 95% confidence interval (CI) = 0.88 ~ 1.12, *P* = 0.85] (Table 1). Similarly, no significant effect was detected by the weighted median method (OR = 0.92, 95% CI = 0.76 ~ 1.13, *P* = 0.45) or the MR-Egger method (OR = 0.91, 95% CI = 0.74 ~ 1.11, *P* = 0.35). Using Cochran Q test, we did not identify any heterogeneity (Cochran Q = 261.26, *P* = 0.12). Besides, test of intercept of the MR-Egger regression showed no significant horizontal pleiotropic effects for the included variants (intercept = 0.002, *P* = 0.31). In addition, leave one out analysis showed that the null IVW estimate was not biased by any single variant. When assessing the causal effect of 25(OH)D on BMI, no evidence of the association between them was observed (IVW β = -0.04, 95% CI = -0.09 ~ 0.01, *P* = 0.15), implying that BMI did not work when considering the causal role of 25(OH)D on OSA.

### Reverse analysis

To further evaluate the causal effect of OSA on circulating 25(OH)D concentration, we conducted a reverse analysis. IVW (six SNPs) suggested a null causal effect of OSA on 25(OH)D (Table 1/Fig. 1). Similar results were observed in other MR models(Table 1). No heterogeneity was shown in Cochran Q test (Q = 9.08, *P* = 0.11), and no horizontal pleiotropy was detected (Intercept = 0.002, *P* = 0.78). However, leave one out analysis indicated that the null estimate was driven by rs10928560 (Fig. S3). After discarding rs10928560, IVW (five SNPs) detected a causal association between OSA and a decreasing concentration of 25(OH)D (β = -0.03, 95% CI = -0.06 ~ -0.01, *P* = 0.01). Similar trends were also shown in other MR methods (Table 1). Again, no heterogeneity or pleiotropy was detected. Leave one out analysis did not detect any points biasing the pooled estimate.

To further evaluate whether this observed causality was driven by BMI, we conducted MVMR analysis to detect the direct effect of OSA on 25(OH)D. Before MVMR analysis, we evaluated the causal role of BMI on 25(OH)D and found that genetic predicted BMI was associated with 25(OH)D (IVW β = -0.10, 95% CI = -0.12 ~ -0.08, *P* = 2.63 × 10^− 23^). This result indicated that genetic predicted obesity was associated with lower 25(OH)D concentration. IVW also detected that genetic predicted higher BMI was causally associated with OSA (IVW OR = 1.74, 95% CI = 1.59 ~ 1.92, P = 1.46 × 10^− 29^). Further MVMR analysis indicated that the observed causal effect of OSA on 25(OH)D was attenuated after accounting for BMI (β = -0.011, 95% CI = -0.028 ~ 0.007, *P* = 0.23).

## Discussion

This bidirectional two-sample MR study evaluating the causality between 25(OH)D and OSA suggested that genetically predicted adult OSA might be causally associated with a lower 25(OH)D concentration. However, no evidence about the causal role of 25(OH)D in OSA was observed. Further MVMR analysis indicated that the causal effect of OSA on 25(OH)D was driven by BMI. To the best of our knowledge, this is the first MR study to explore the causality between 25(OH)D and OSA.

Previous observational studies evaluating the association between 25(OH)D and OSA yielded inconsistent results. Some studies reported a significant relationship between a lower level of serum 25(OH)D and OSA. Goswami and colleagues found the relationship between OSA and 25(OH)D insufficiency may be mostly attributed to higher BMI and bigger neck circumference [20]. According to Upala and Sanguankeo’s preliminary review on 506 subjects, those with OSA had lower 25(OH)D levels compared to controls [β (95% CI): − 5.81 (-10.09, -1.53, *P* = 0.008)[21]. Some studies also reported a decreasing 25(OH)D level with increasing OSA severity[10, 11, 22]. However, some studies found no relationship between 25(OH)D and OSA. For example, Salepci et al. found that the percentage of 25(OH)D deficiency is similar in participants with or without OSA [23]. Considering that traditional observational studies could not fully mitigate confounders or reverse causality, the causal association between 25(OH)D and OSA warrants further investigation.

Using MR design, this study provided evidence about the causal impact of OSA on a decreasing 25(OH)D level. The potential mechanisms underlying this causality are worth discussing. Specifically in the present study, MVMR indicated that the causal effect of OSA on 25(OH)D might be driven by BMI. This might have an implication in clinical practice that obesity management should be reinforced in patients with OSA to prevent 25(OH)D deficiency. Based on the existing literature, obesity has been identified as a major risk factor for OSA. Studies have confirmed that the excess of fat deposition surrounding the airway contributes to the narrowing of upper air way[24, 25]. Besides, neuroanatomic interactions can be impaired by obesity-related leptin resistance, which is involved in the genesis of OSA [26]. Despite of this, we caution that these findings require validation in clinical intervention studies to determine the exact effects. Except for the mechanisms described above, previous studies have also reported some evidence of the links between OSA and 25(OH)D. As known, vitamin D deficiency can be caused by a variety of factors, like geographical location, and solar exposure[27]. In terms of the potential pathways linking OSA to a lower 25(OH)D level, sleep fragmentation might be a reasonable explanation. Sleep fragmentation caused by nocturnal hypoxia in people with OSA has been related to daytime drowsiness, exhaustion, and other symptoms, consequently leading to a lower frequency of outdoor activity and reducing vitamin D synthesis[28]. Besides, because of the frequent exacerbation of upper airway obstruction and hypopnea for OSA patients, autonomic nerve function is mostly increased in terms of sympathetic nerve activity at night, and the vagus nerve is abnormally tense[29]. The sympathetic nervous system’s stimulation may partially suppress vagus nerve activity, whereas aberrant vagus nerve activity affects gastrointestinal motility and the release of gastrointestinal hormones, which impacts vitamin D absorption and metabolism. OSA patients have long experienced sleep disorders, intermittent hypoxia, elevated abdominal pressure, and upper airway obstruction[30]. All of these conditions can lead to gastroesophageal reflux and stomach ischemia, which can impair vitamin D absorption[31].

Our research made several signs of progress. First, to the best of our knowledge, no MR study on the relationship between 25(OH)D and OSA has yet been explored. To enhance the statistical power to detect causal relationships, we used numerous variants summarized from large-scale GWAS research on 25(OH)D and OSA to date. Second, using the MR design, our study simulated RCTs in observational settings. RCTs are regarded as the golden standard for causal inference, but the high cost makes it impractical for many scholars. MR studies can effectively avoid confounding bias because SNPs were randomly assigned at conception. Compared to other observational studies, MR can also avoid the reverse causal effect. Third, a crucial strength of our research, compared to the analysis of individual-level data from a small study, is that effect-size data were gathered from large-scale GWASs for exposure and outcome traits, allowing us to quantify effect sizes more precisely.

There are several limitations to our research. First, although limiting the sample to people of European ancestry reduced population structure bias, this may restrict the generalizability of our findings to other populations. Second, population stratification could not be fully ruled out in our study. One point that should be noted is that our OSA GWAS data was derived from participants from Finland, a northern European country with low sun exposure. Therefore, national program specific to this county, like Vitamin D fortification in milk products, could potentially influence the standard 25(OH)D levels in Finland population, which might induce bias to our MR results. Third, our findings only reported the total effect of OSA on 25(OH)D concentration, but the underlying mechanisms warrant further investigation. Firth, only summary-level statistics were used in this study, making stratification analysis like severity, BMI, gender or age stratification unavailable. However, the original GWAS analysis from the FinnGen research project has adjusted for sex and age. More detailed MR analyses are warranted when GWAS with severity distribution is publicly available in the future. In addition, considering the critical role of BMI in OSA development, the present study used MVMR to adjust with BMI to detect the direct effect of OSA on 25(OH)D.

In conclusion, this MR study showed that genetically predicted OSA decreased circulating 25(OH)D levels, which might be driven be BMI. These results provided a novel insight into the role of OSA in 25(OH)D metabolism and might have an implication for clinicians to enhance obesity management among the patients with OSA in clinical practice to prevent 25(OH)D deficiency, as that 25(OH)D is widely involved in multiple health conditions.

## Electronic supplementary material

Below is the link to the electronic supplementary material.

Supplementary Material 1

Supplementary Material 2

Supplementary Material 3

## Data Availability

The Additional file contains all of the exposure SNPs that were used. They can also be found online in the MRC IEU repository, which is accessible at https://gwas.mrcieu.ac.uk.ieu-b-4808,finn-b-G6 SLEEPAPNO.

## Abbreviations

25(OH)D: 25-hydroxyvitamin D
OSA: Obstructive Sleep Apnea
MR: Mendelian randomization
IVW: inverse variance weighted
RCTs: randomized controlled trials
SNPs: single nucleotide polymorphisms
IVs: instrumental variables
GWAS: genome-wide association study
LD: linkage disequilibrium
BMI: body mass index
OR: odds ratio
CI: confidence interval
MVMR: Multivariate Mendelian randomization
