# Supplementary material for: Bi-directional and multivariate mendelian randomization analysis of the relationship between circulating 25-hydroxyvitamin D concentration and obstructive sleep apnea
Source: BMC Pulm Med. 2022 Oct 14;22:380. doi: 10.1186/s12890-022-02172-y (PMC9569054; doi:10.1186/s12890-022-02172-y)
Supplement: Supplementary file 3 — Supplementary Material 3 [file 12890_2022_2172_MOESM3_ESM.pdf]

Supplementary Materials

Bi-directional Mendelian Randomization Analysis of the Relationship between Circulating 25-hydroxyvitamin D Concentration and Obstructive Sleep Apnea Risk

Primary commands in current study

Table S1. Harmonization data of 25(OH)D on OSA.

Table S2. Harmonization data of OSA on 25(OH)D.(Sheet 1 is the harmonization after discarding rs10928560; Sheet 2 is the harmonization before discarding rs10928560)

Figure S1. Scatterplot of bidirectional Mendelian randomisation analyses [(A) Scatterplot of 25OHD-OSA risk MR; (B) Scatter plot of OSA risk-25OHD MR;(C) Scatterplot of OSA risk-25OHD MR after discarding rs10928560].

Figure S2. Forest plot of bidirectional Mendelian randomisation analyses [(A) Forest plot of 25OHD -OSA risk MR; (B) Forest plot of OSA risk-25OHD MR; (C) Forest plot of OSA risk-25OHD MR after discarding rs10928560].

Figure S3.(A)MR leave−one−out sensitivity analysis for '25OHD' on 'OSA'; (B) MR leave−one−out sensitivity analysis for 'OSA on '25OHD ';(C) MR leave−one−out sensitivity analysis for 'OSA on '25OHD ' after discarding rs10928560].

Figure S4.Funnel plot of 25OHD-OSA risk MR

A

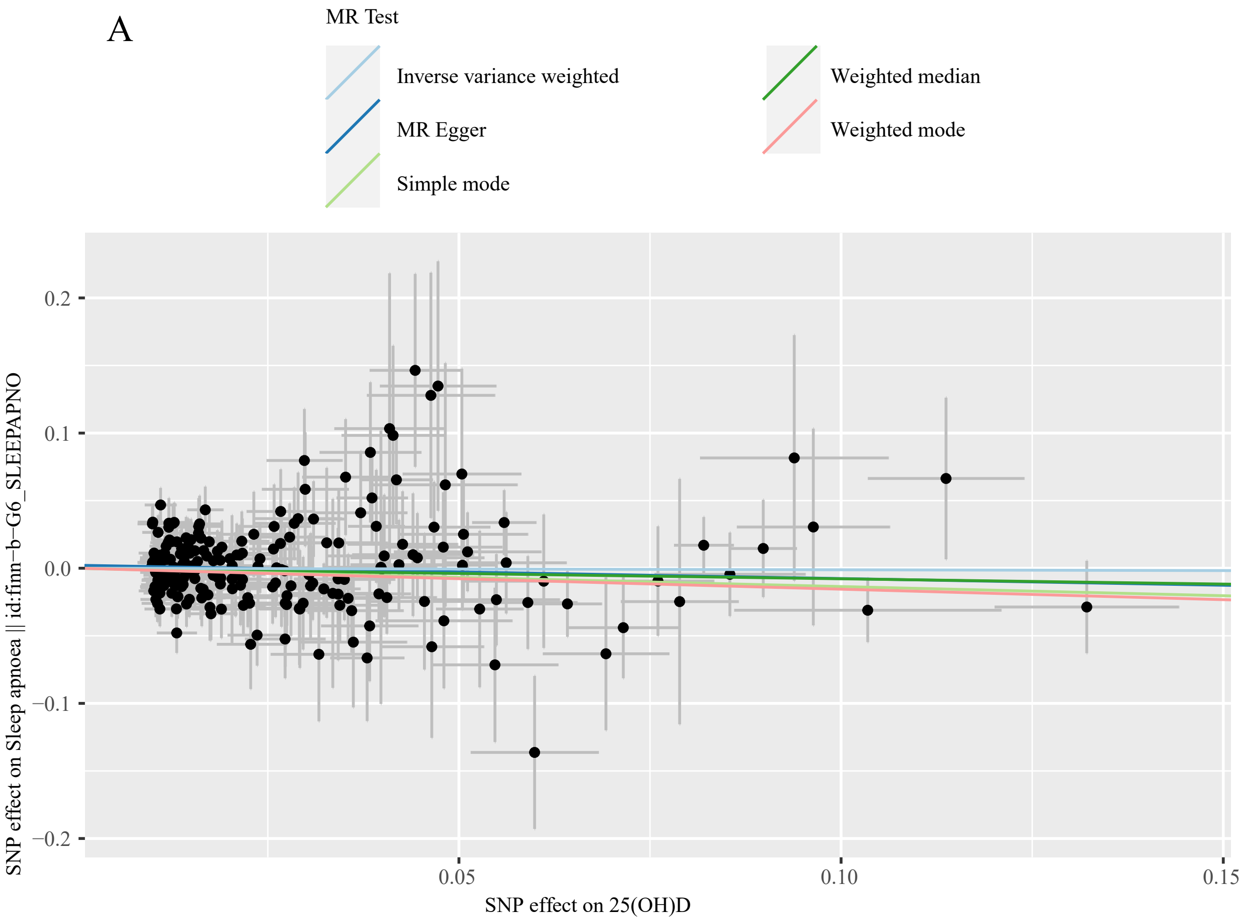

MR Test
Inverse variance weighted
MR Egger
Simple mode
Weighted median
Weighted mode
0.2
0.1
0.0
−0.1
−0.2
SNP effect on Sleep apnoea || id:finn−b−G6_SLEEPAPNO
0.05
0.10
0.15
SNP effect on 25(OH)D

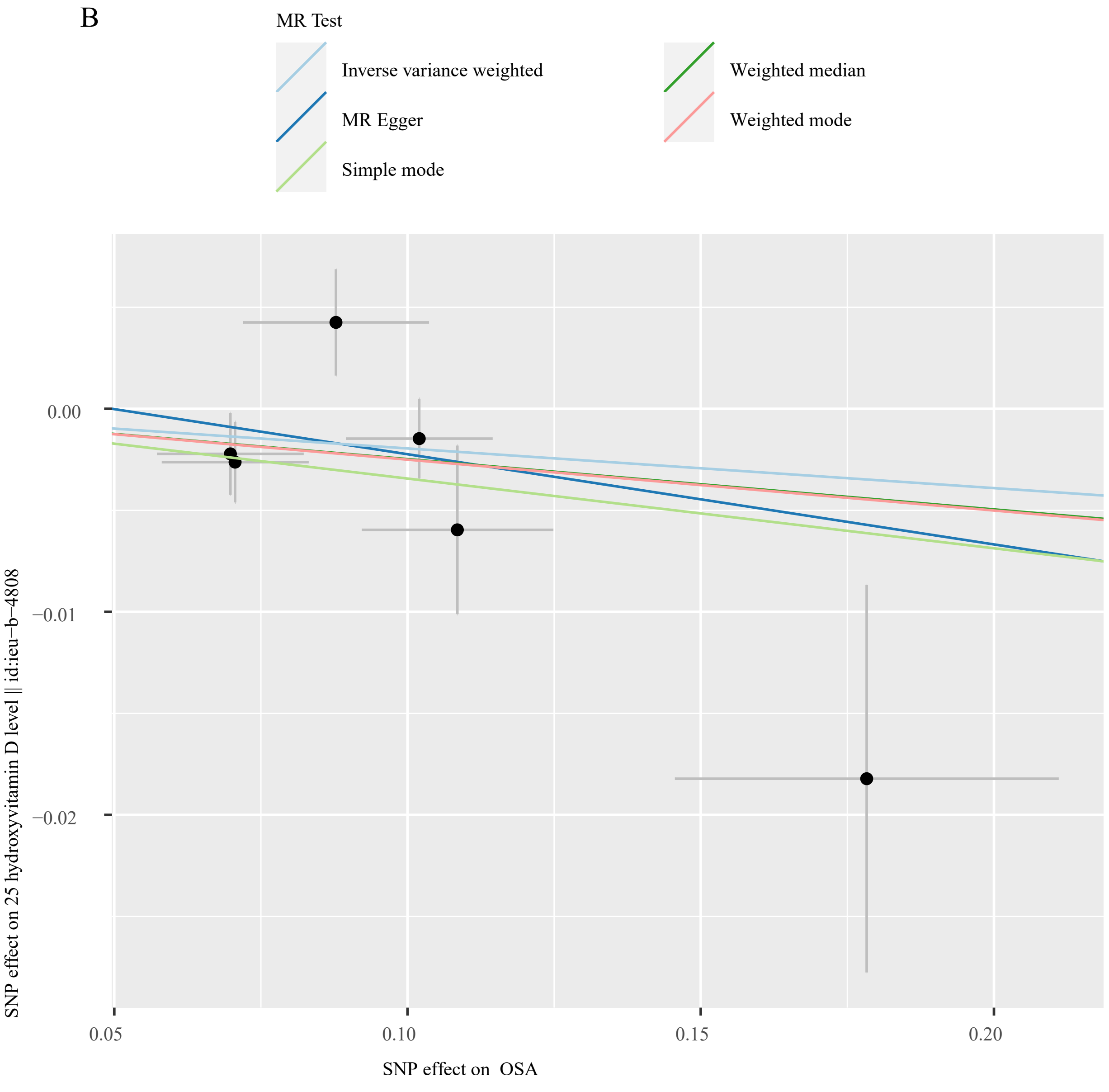
B
MR Test
Inverse variance weighted
MR Egger
Simple mode
Weighted median
Weighted mode
0.00
−0.01
−0.02
SNP effect on 25 hydroxyvitamin D level || id:ieu−b−4808
0.05
0.10
0.15
0.20
SNP effect on OSA

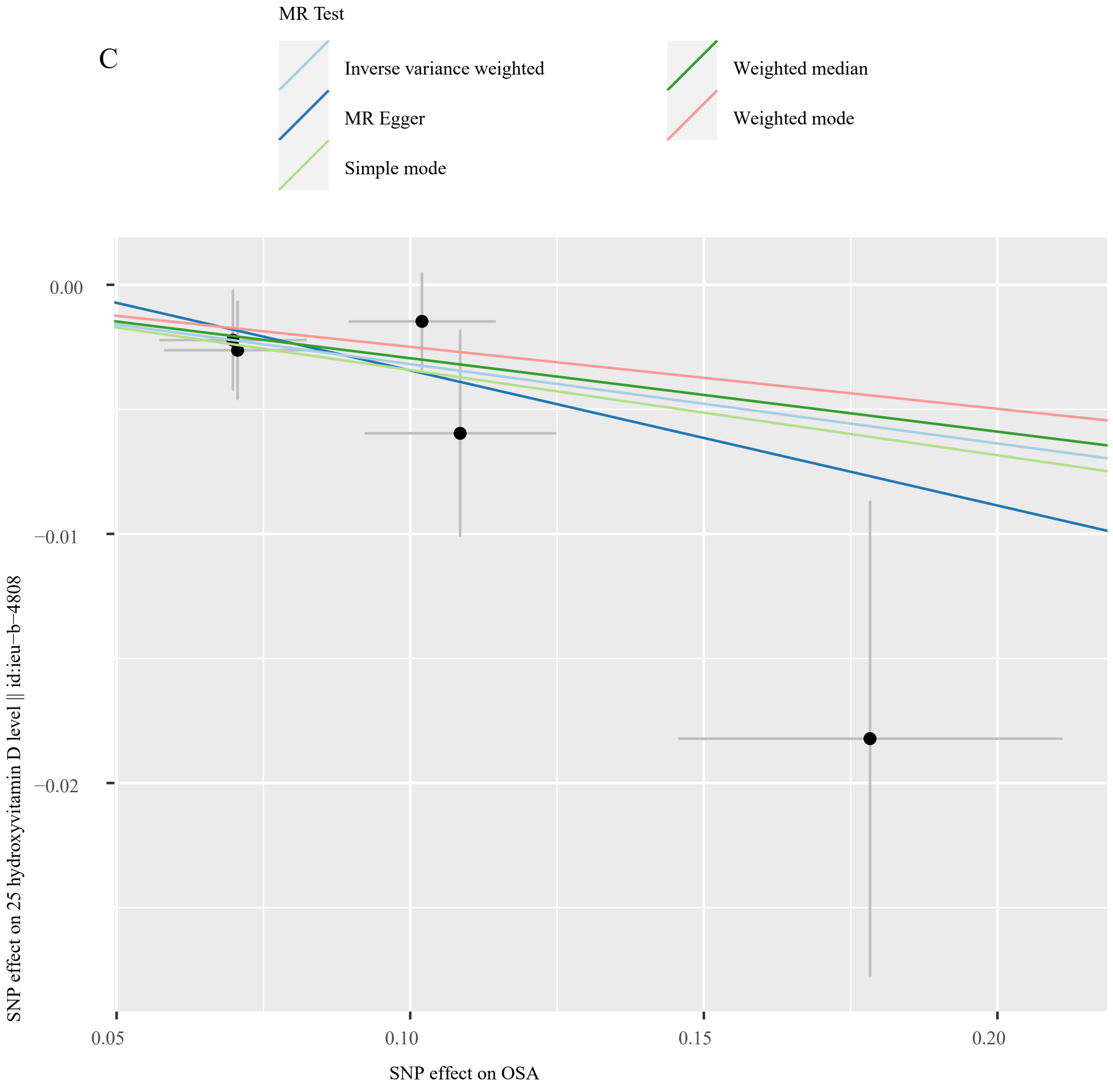

FIGURE S1

Scatterplot of bidirectional Mendelian randomisation analyses [(A) Scatterplot of 25OHD -OSA risk MR; (B) Scatter plot of OSA risk-25OHD MR;(C) Scatterplot of OSA risk-25OHD MR after discarding rs10928560].

A

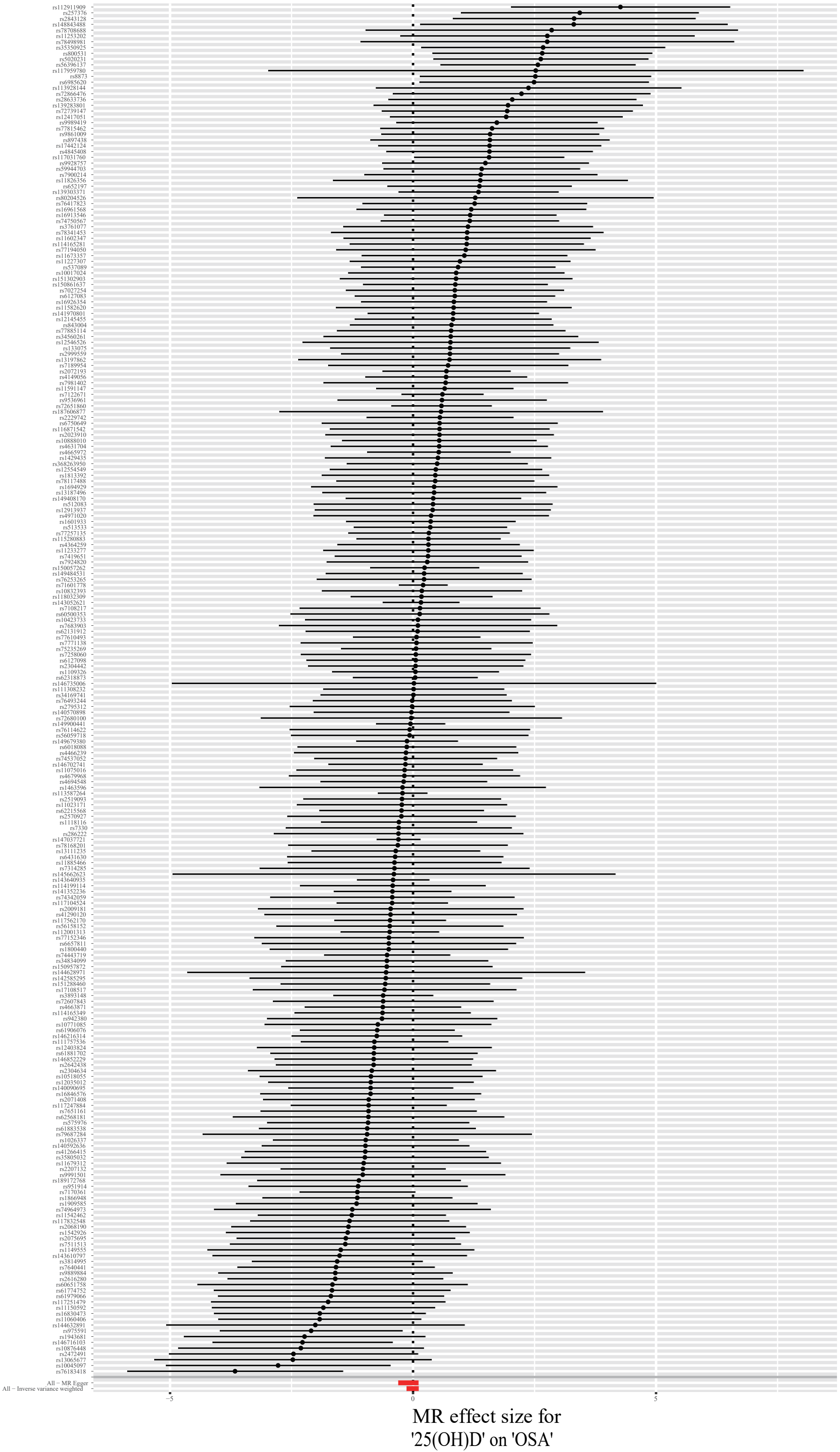

All – MR Egger
All – Inverse variance weighted
-5
0
5
MR effect size for
'25(OH)D' on 'OSA'

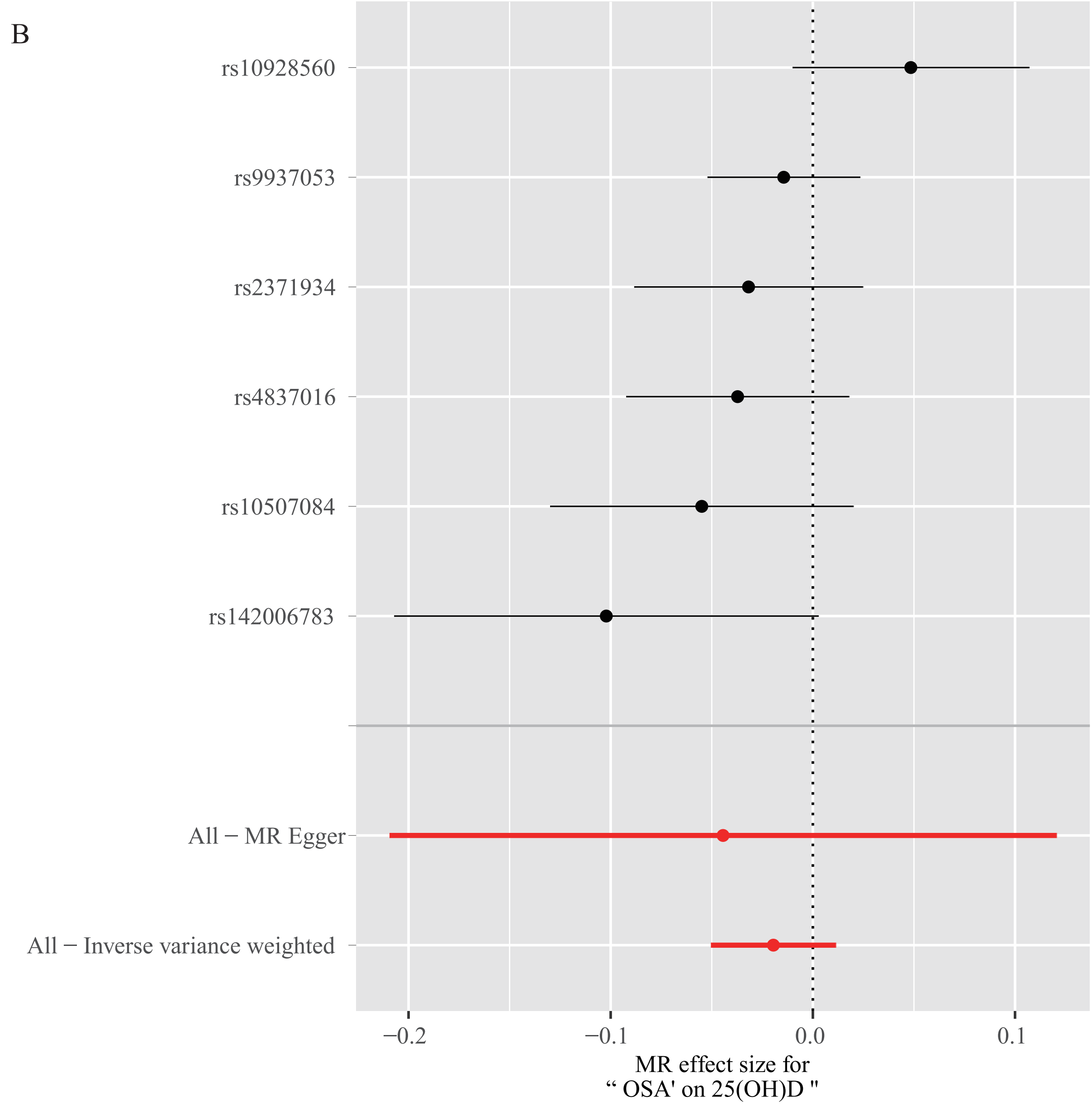
B
rs10928560
rs9937053
rs2371934
rs4837016
rs10507084
rs142006783
All − MR Egger
All − Inverse variance weighted
−0.2
−0.1
0.0
0.1
MR effect size for
“ OSA' on 25(OH)D "

C

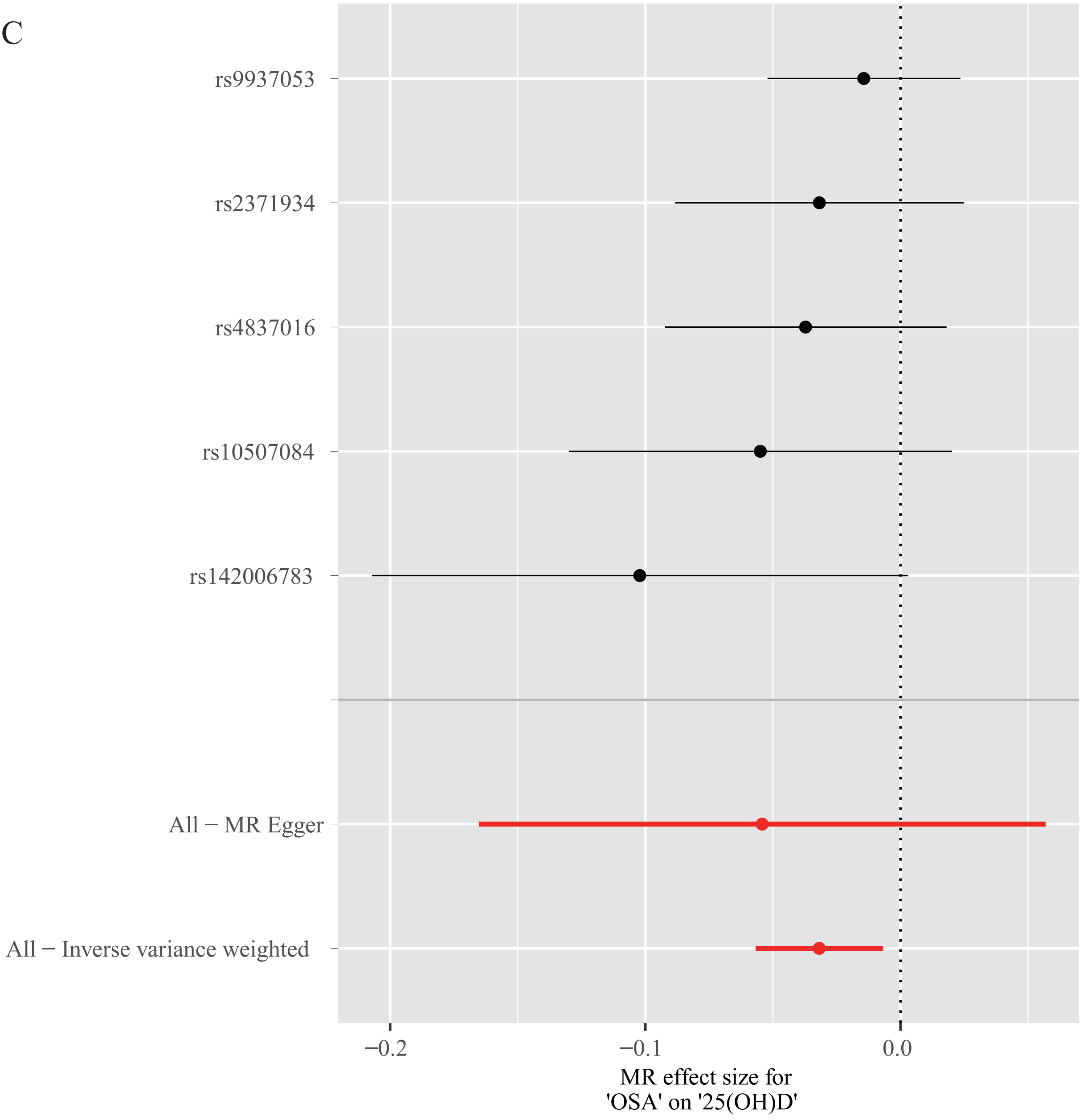

FIGURE S2
Forest plot of bidirectional Mendelian randomisation analyses [(A) Forest plot of 25OHD -OSA risk MR; (B) Forest plot of OSA risk-25OHD MR; (C) Forest plot of OSA risk-25OHD MR after discarding rs10928560]..

A

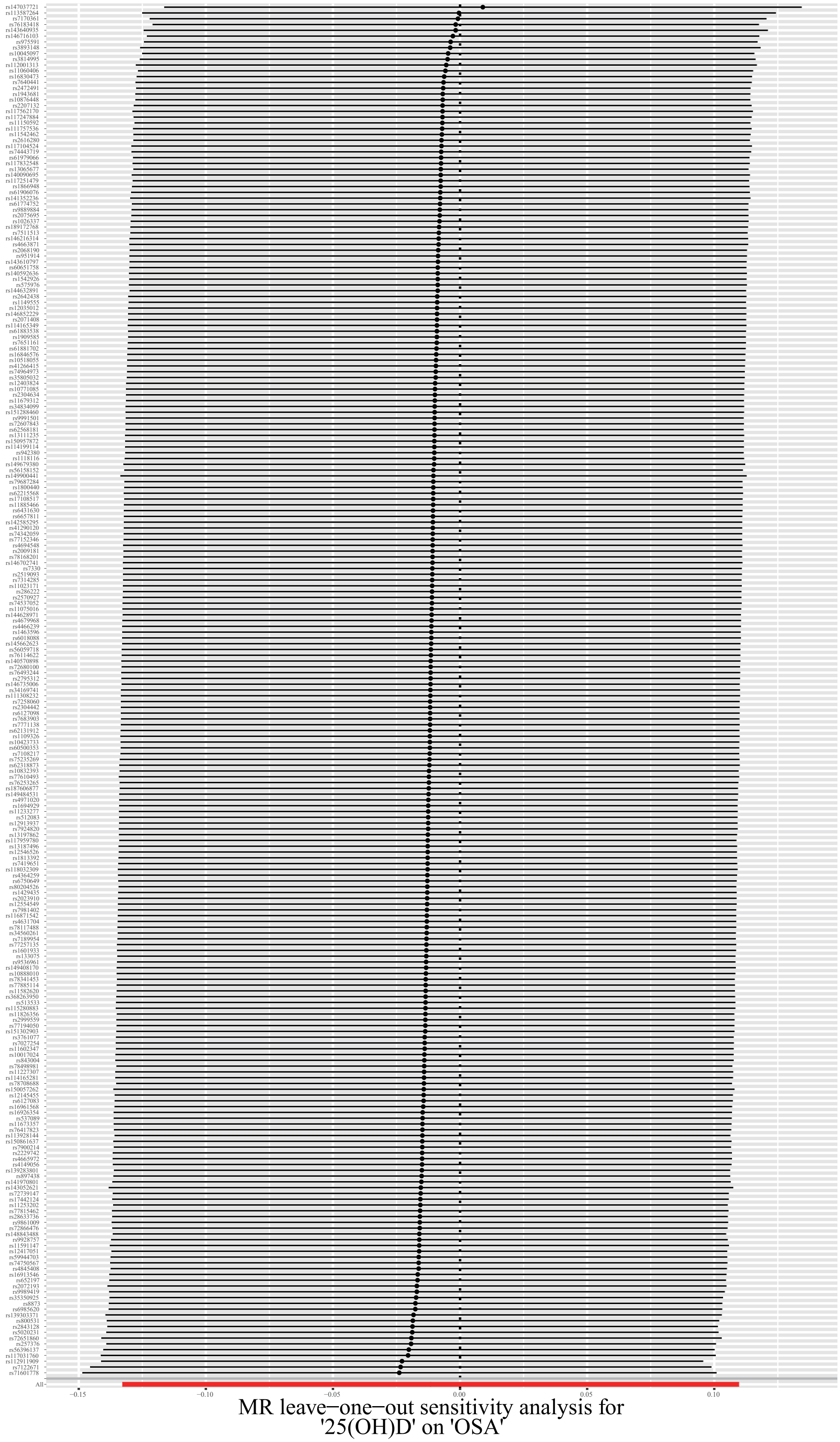

All
−0.15
−0.10
−0.05
0.00
0.05
0.10
MR leave−one−out sensitivity analysis for
'25(OH)D' on 'OSA'

B

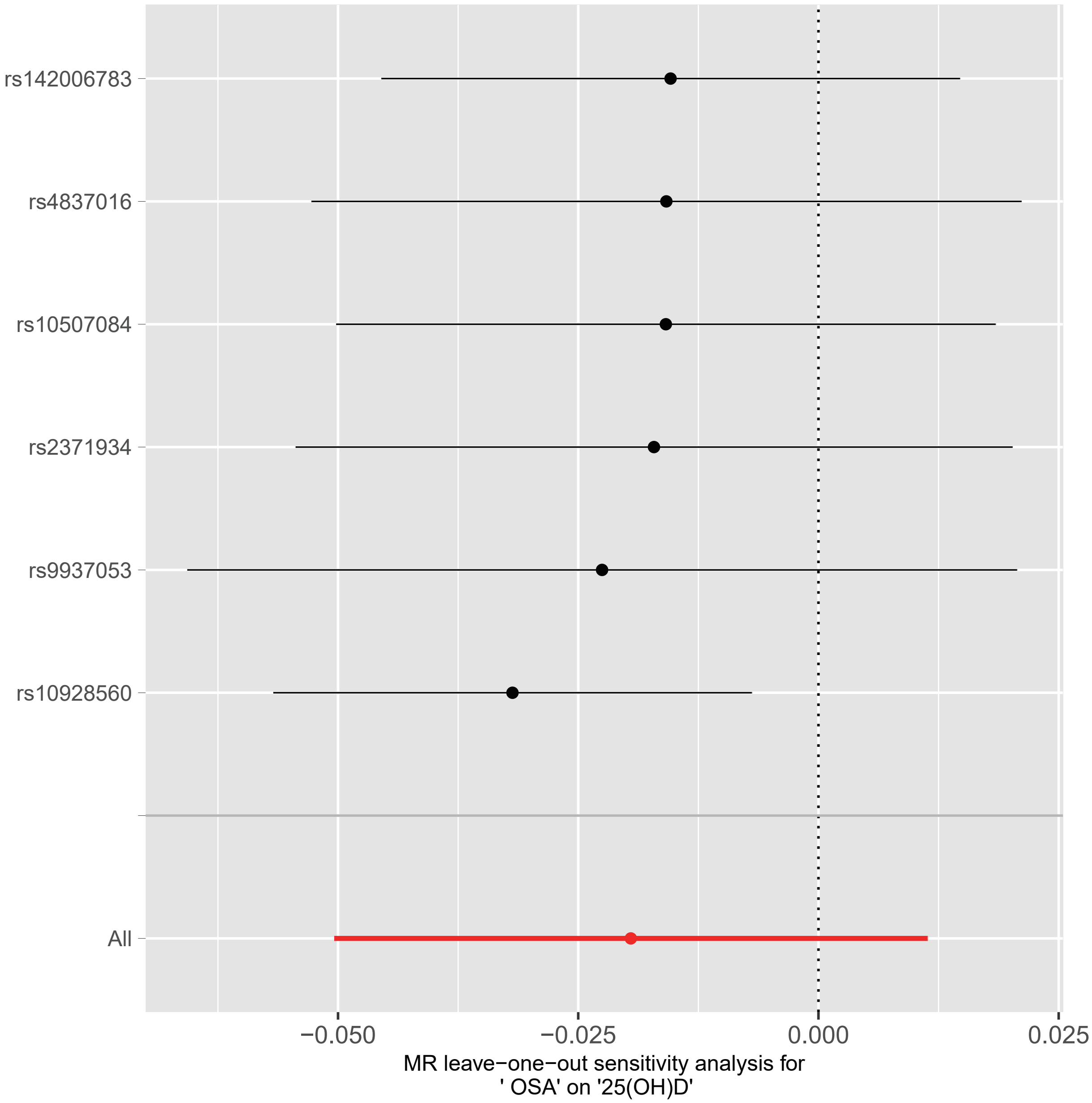
rs142006783
rs4837016
rs10507084
rs2371934
rs9937053
rs10928560
All
−0.050
−0.025
0.000
0.025
MR leave−one−out sensitivity analysis for
' OSA' on '25(OH)D'

C

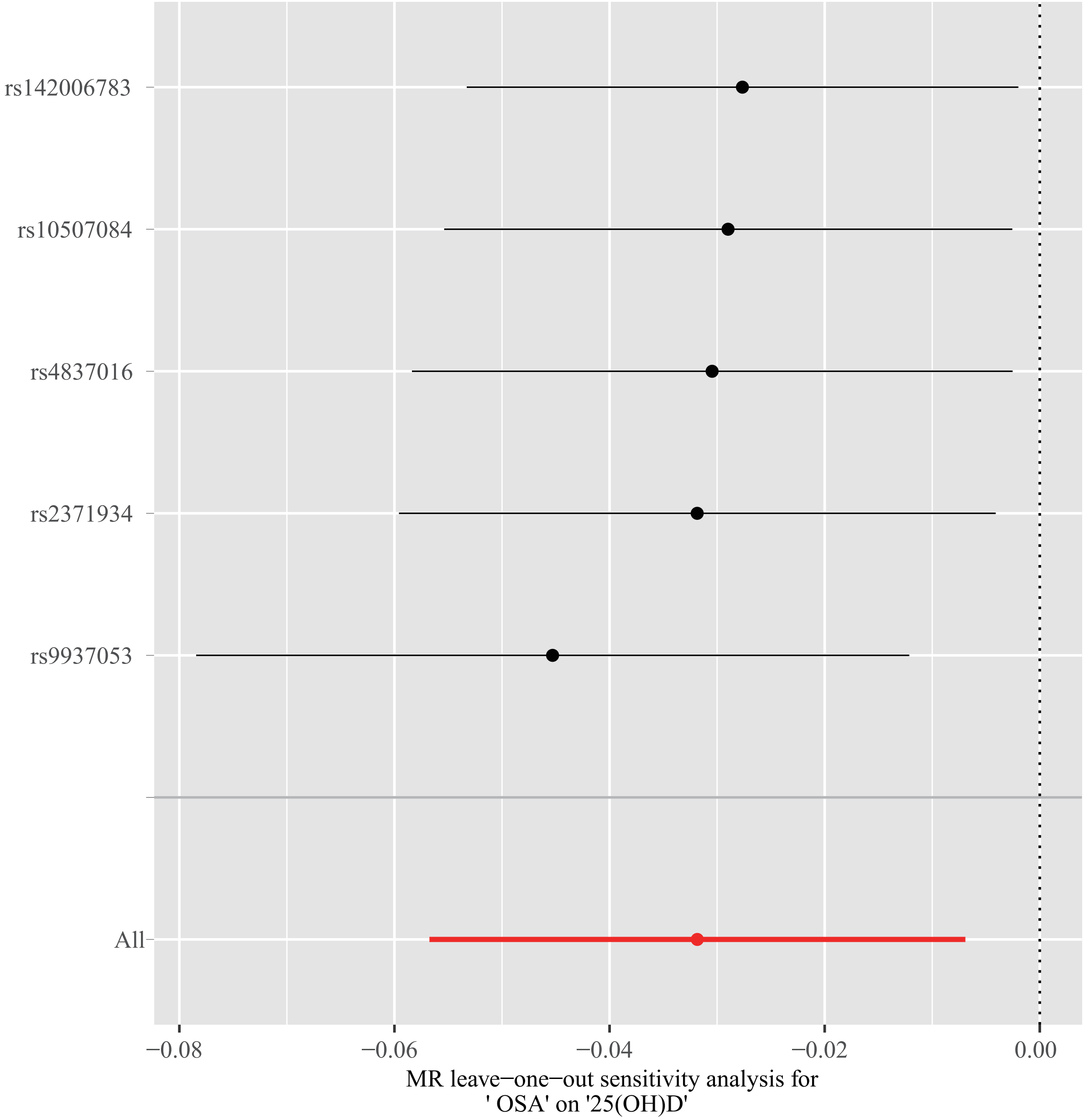

FIGURE S3
.(A)MR leave−one−out sensitivity analysis for '25OHD' on 'OSA'; (B) MR leave−one−out sensitivity analysis for 'OSA on '25OHD ';(C) MR leave−one−out sensitivity analysis for 'OSA on '25OHD ' after discarding rs10928560].

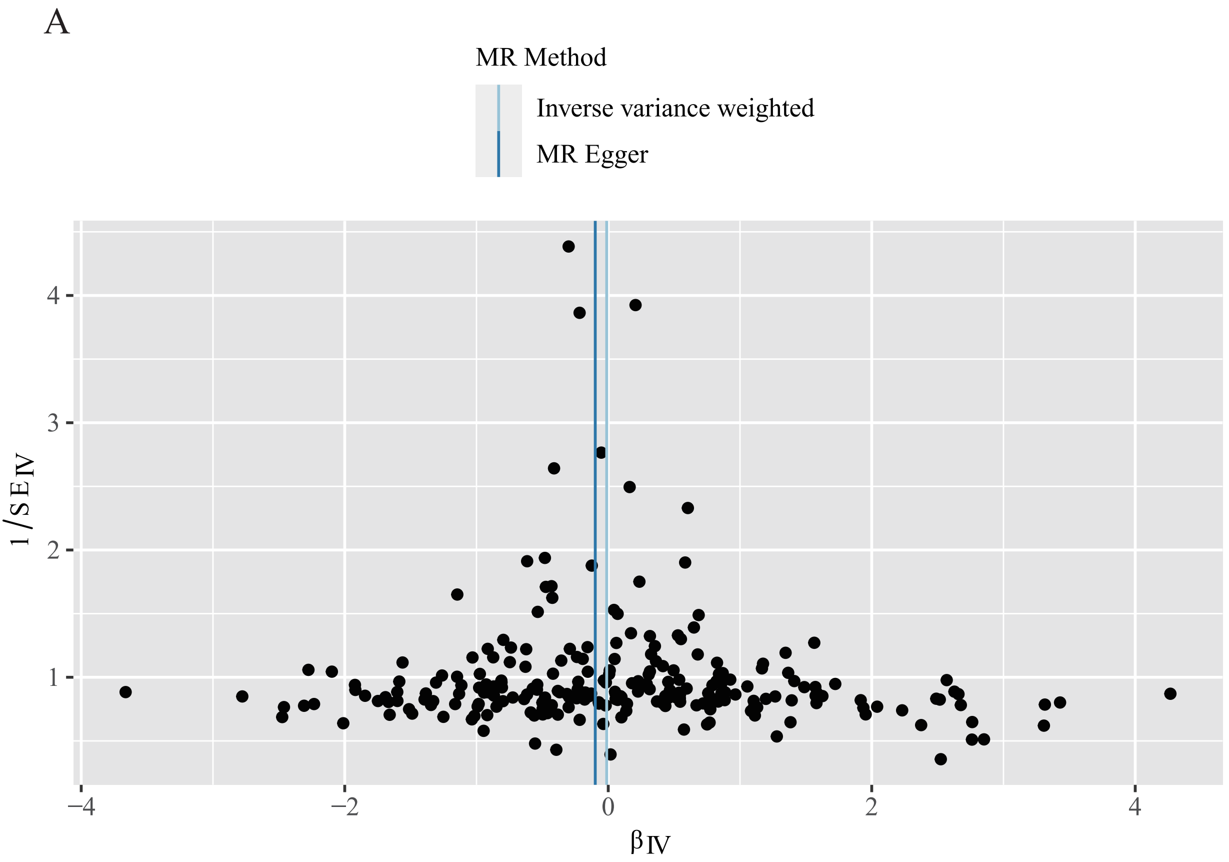

FIGURE S4
Funnel plot of 25OHD -OSA risk MR.
